# Retirement from elite sport and self-esteem: a longitudinal study over 12  years

**DOI:** 10.3389/fpsyg.2023.1176573

**Published:** 2023-05-05

**Authors:** Jürg Schmid, Achim Conzelmann, Robertino Engel, Andreas Kuettel, Michael J. Schmid

**Affiliations:** ^1^Institute of Sport Science, University of Bern, Bern, Switzerland; ^2^Department of Sports Science and Clinical Biomechanics, University of Southern Denmark, Odense, Denmark

**Keywords:** athletic retirement, career transition, elite sport, self-esteem, longitudinal study

## Abstract

This study examined the complex associations between athletic retirement and self-esteem among former elite athletes. With reference to theoretical and empirical work on the quality of the transition out of sport, information was collected from 290 (junior) elite athletes in a retrospective-prospective design: at the first measurement, active athletes assessed satisfaction with their sporting career, athletic identity, and self-esteem. At the second measurement (12  years later), the now former athletes rated transition characteristics of their career ending, sporting career success, emotional reactions to career termination, extent of necessary adjustment required following athletic retirement, duration and quality of adjustment, and self-esteem. Structural equation modelling revealed that neither sporting career success nor sporting career satisfaction had a direct effect on adjustment. However, athletic identity and retirement planning predicted the extent of adjustment, which in turn predicted the duration and quality of adjustment, and ultimately self-esteem. Voluntariness, timeliness, and perceptions of gain predicted emotional reactions towards career termination, which also predicted the duration of adjustment. Extent of adjustment and emotional reactions mediated between preconditions of career termination and transition characteristics and self-esteem. While self-esteem after career termination was predominantly predicted by self-esteem 12  years earlier, perceived quality of adjustment to career termination had a significant effect on self-esteem in the post-athletic career. These results complement existing literature illustrating that athletic retirement is a complex and dynamic process and the quality of this transition has a small, but still noteworthy effect on self-esteem, a central construct for well-being.

## 1. Introduction

For many elite athletes, sport represents a (quasi-)professional activity which is oriented towards athletic excellence and sporting success. Professionalized sporting careers require athletes to prioritize time for training and competition, consequently subordinating other interests. Moreover, many athletes develop a strong athletic identity, and their sport achievements and social recognition contribute substantially to their self-esteem (Alfermann and Stambulova, 2007).

Once the athletic career comes to an end, athletes are often challenged with major changes in several spheres of live (Stambulova and Samuel, 2020), including occupational, financial, social, and bodily changes (Stephan et al., 2003a; Cecić Erpič et al., 2004). Although these changes may present risks as well as opportunities, existing literature focuses on the negative effects of athletic retirement (Knights et al., 2016). Often cited as a source of distress is the socio-professional transition, an athlete’s transition from familiar, acclaimed and highly skilled (quasi-)professional activity in sports to a new occupational activity in a different context, a descent from the heights of the extraordinary into the flats of ordinariness (Sparkes, 1998). Former athletes are faced with the demands of a novel work environment and have to build new skills and competencies for their new jobs in which they may never reach a comparable feeling of competence and self-efficacy or receive the same amount of public attention and recognition (Stephan and Demulier, 2008; Park et al., 2013).

Well recognized in the literature is the notion of transition as an event that, according to Schlossberg (1981, p. 5), involves the change of “assumptions about oneself and the world and thus requires a corresponding change in one’s behavior and relationships” (see also Wylleman, 2019). Unsurprisingly, athletic retirement has also been found to be associated with changes in self-esteem (Stambulova, 2017). However, only a small number of empirical studies have investigated the effects of athletic career transitions on self-esteem, especially when it comes to athletic career termination.

Conversely, self-esteem defined as “an individual’s subjective evaluation of his or her worth as a person” (Orth and Robins, 2019, p. 329), has attracted considerable research interest in developmental psychology, because it has been shown to predict or at least to be associated with numerous important outcomes. Related outcomes include satisfaction in marriage and close relationships, size of social-network and support, physical and mental health, educational and occupational achievement, employment status, and job satisfaction (Orth and Robins, 2014). Both theoretical and empirical research have suggested that major life events and transitions, for example, parenthood, community relocation, and workforce retirement, can induce change in self-esteem (e.g., Kling et al., 2003; Bleidorn et al., 2016; Bleidorn and Schwaba, 2018).

Among the few studies in sport psychology on athletic retirement and self-esteem is one by Cecić Erpič et al. (2004). They found that athletes experienced various psychological difficulties during the process of sports career termination such as feelings of incompetence in activities other than sports and lowered self-respect, self-confidence, and self-esteem. Particularly affected were former athletes who had retired involuntarily, had achieved fewer goals than expected, and identified strongly with the athletic role (Cecić Erpič et al., 2004). Similarly, Kleiber and Brock (1992) found that 5 to 10 years after athletic retirement, former elite college athletes who had sustained a career-ending injury and have been highly involved in sports reported lower self-esteem than former athletes who had been less involved and had not suffered from such an injury.

Studies comparing groups of still active and retired athletes, however, support only partially these results: Gouttebarge et al. (2015) found that 5% of former professional footballers scored below the threshold indicating the presence of adverse self-esteem, whereas the corresponding prevalence among current professional footballers was 3%. In contrast, Marin-Urquiza et al. (2018) did not detect any differences in self-esteem, while Shaffer (1990) found that retired athletes scored higher on self-esteem than still active ones, a result that was attributed to the fact that retired athletes are no longer subjected to the judgmental atmosphere of competitive sport.

Two longitudinal studies yielded more instructive results. Particularly, Stephan et al. (2003a) investigated the self of 16 active elite athletes and 16 elite athletes transitioning out of sport four times over a one-year period. Compared to active athletes, transitional athletes had a lower physical self and lower global self-esteem at all measurement points. Importantly, physical self-worth and global self-esteem decreased during the first 6 months of transition out of elite sport. This initial period was followed by a period of adaptation, in which athletes reassessed their physical competencies and personal standards, resulting in the stabilization of these constructs.

In reviewing the literature, one finds only a few studies addressing self-esteem in the post-athletic life, and for methodological reasons it is unclear to what extent their findings are reliable. Specifically, the cited studies draw either upon subjective accounts regarding temporal changes in self-esteem, upon retrospective measurements of self-esteem, or upon still active athletes as controls. Furthermore, sample sizes were small in the studies using a longitudinal design, which is clearly called for in this field of research.

The present study assumes that major life events such as athletic career termination affect self-esteem. This assumption rests on both research in life-span developmental psychology on workforce retirement (e.g., Bleidorn and Schwaba, 2018), career research in sport (e.g., Debois et al., 2015), and mental health research in elite sport. In particular, Turner (2020) argued that poor mental health and, we might add, its correlates such as low self-esteem (Sowislo and Orth, 2013) are likely to depend on how an athlete’s career ends. Similarly, Kuettel and Larsen (2020) identified in their scoping review a “successful retirement adjustment” as a major protective factor for former elite athletes’ mental health.

Understanding “successful retirement adjustment” and its contributing factors, is the aim of several theoretical models of athletic career termination and of career transitions (for a review, see Stambulova and Samuel, 2020). However, these models are conceptual in nature and do not lend themselves to formulating testable hypotheses about factors contributing to the quality of the transition out of sport and self-esteem. Instead, we drew on three systematic reviews (Park et al., 2013; Knights et al., 2016; Barth et al., 2021), examining the influence of various individual, situational, and contextual factors on the adjustment to athletic retirement, to hypothesize the model.

Therefore, the purpose of the present study was to provide a more complete understanding of how the transition out of elite sport is associated with interindividual differences in self-esteem in life after elite sport. In particular, we wanted to investigate how several (individual) preconditions of the career termination and transition characteristics are associated with the duration and quality of adjustment to athletic retirement, and ultimately self-esteem. To do this, we adopted a holistic lifespan perspective and used mixed retrospective-prospective longitudinal data from a sample of Swiss elite athletes. We developed a structural equation model (SEM) which will be presented in the next section. The specific aim of the present study was to test this model and to improve on the model, if empirical evidence suggests doing so.

**Figure 1:**
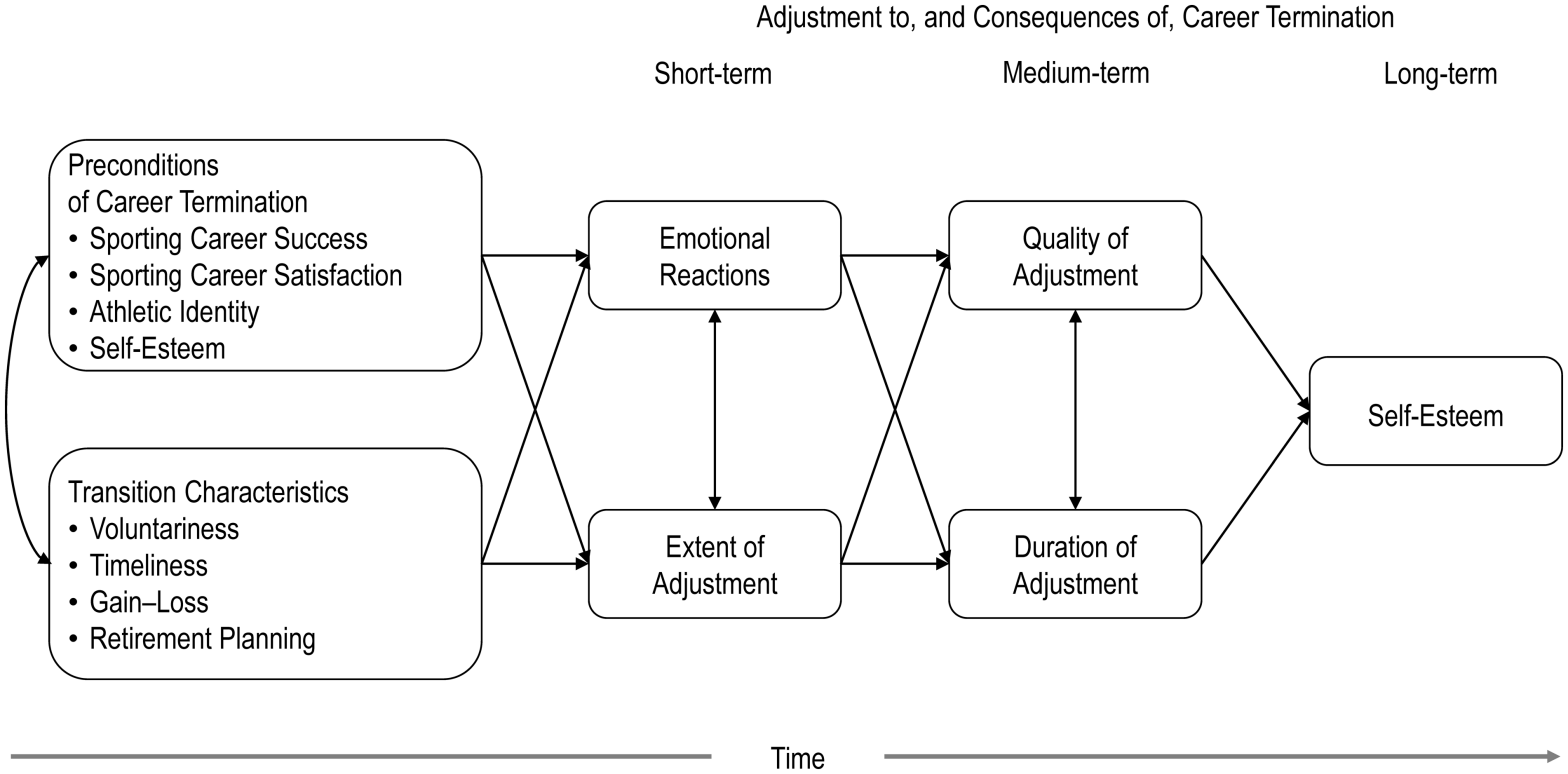
Schematic overview over the hypothesized model.

The core elements of the hypothesized model are shown in Figure 1. Based on empirical findings, the model posits that preconditions of career termination and transition characteristics predict emotional reactions to career termination and the extent of necessary adjustment to life thereafter. Further, we hypothesize that these short-term effects of career termination predict two medium-term consequences, that is the duration and quality of the adjustment process, which in turn shape the long-term adjustment and consequences represented by self-esteem.

**Figure 2:**
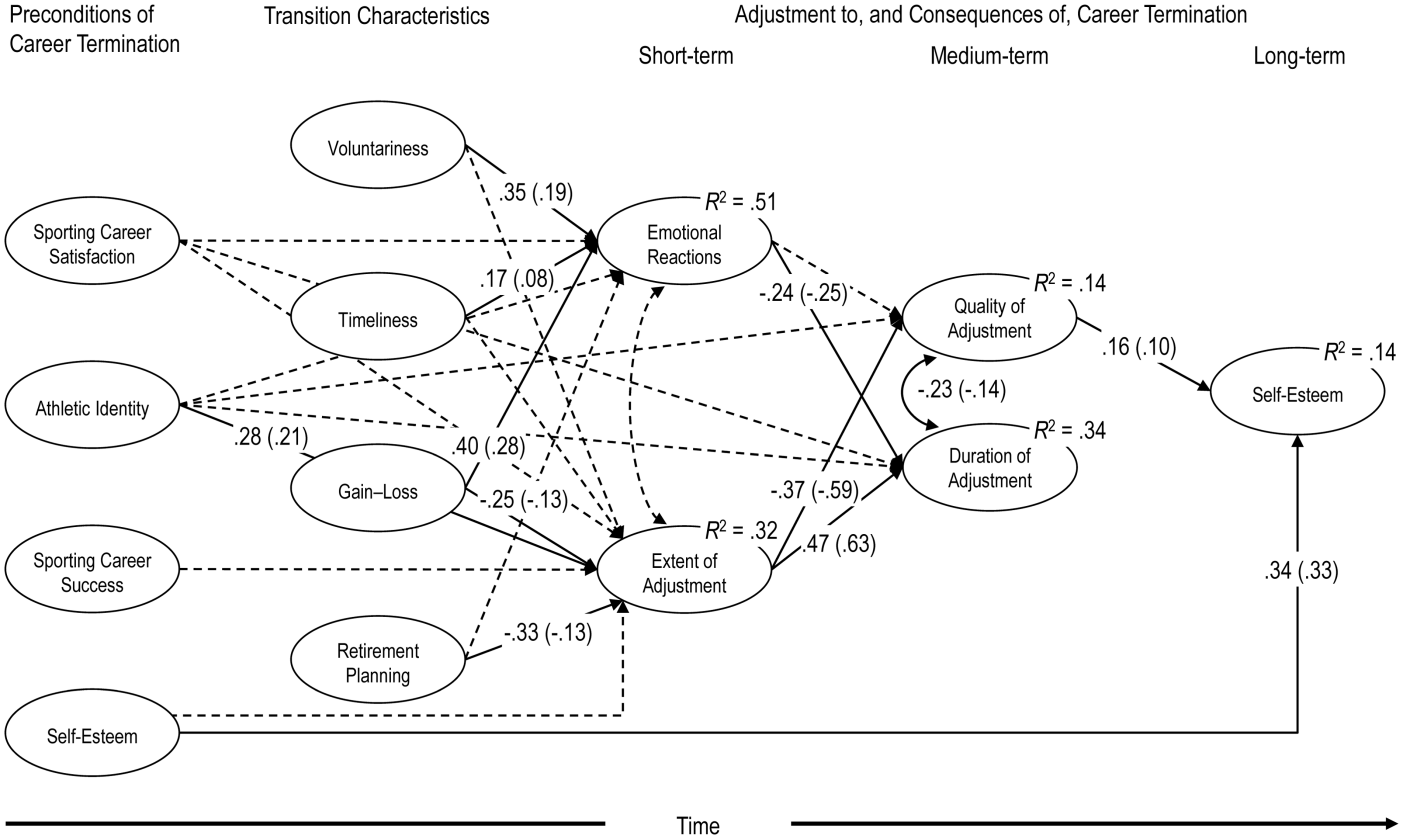
Structural equation model predicting self-esteem after athletic career termination.

Figure 2 illustrates the hypothesized model, completed with parameter estimates due to space limitations. Looking at preconditions of career termination, we expected objective sporting career success (i.e., taking part successfully in major international competitions), which is often linked with a secured income and potential for savings, to influence the extent of adjustment in that the availability of financial resources reduces the immediate pressure to adjust in financial and vocational regard (Alfermann and Stambulova, 2007). Because research has shown that a sense of achievement in one’s athletic career can facilitate the transition into life beyond sport (Cecić Erpič et al., 2004; Barriopedro et al., 2019), we assumed positive effects of sporting career satisfaction, that is the evaluation of one’s athletic career, on the emotional reactions. Drawing on coping research, which emphasizes that positive attitudes towards oneself are a valuable coping resource (Hoar et al., 2006), it was also hypothesized that career satisfaction is negatively linked to the extent of adjustment required following career termination. Based on Cecić Erpič et al. (2004), we expected sporting career satisfaction to be directly linked with the duration of adjustment: it might be difficult to close a chapter and move on with life, when one’s athletic career turned out to be less successful or satisfying than hoped for (Kleiber et al., 1987).

A strong or exclusive athletic identity has typically been found to increase professional, social, and emotional difficulties (Ronkainen et al., 2016; Kuettel et al., 2017), increase the time necessary to adjust to life after sport (e.g., Park et al., 2013), and strain the quality of adjustment in general (Lally, 2007). We expected that athletic identity would influence the duration of adjustment directly and indirectly through an effect on emotional reactions and the extent of adjustment (Samuel et al., 2019). However, we did not presume a long-term effect on self-esteem because athletic identity was found to be unrelated to a similar construct, life satisfaction (Shander and Petrie, 2021).

The addressed preconditions of career termination are set at a general level, where both career success and career satisfaction span the entire sporting career. By comparison, the transition characteristics are located on a more specific level because they refer to a particular event in time, namely the ending of an athletic career. We expected them to have short-term effects, but not to be directly related to medium- or long-term consequences (e.g., self-esteem).

Four transition characteristics, all of which have been consistently found to be relevant in previous studies, were included in the model: (a) voluntariness, (b) timeliness of an athlete’s career end, (c) perceptions of gain vs. loss regarding career termination, and (d) planning for athletic retirement and post-career life. Planning refers to having clear plans for the future and preparing for career termination, and was expected to foster positive emotional reactions upon retirement and reduce the nature and extent of adjustment (Alfermann et al., 2004; Stambulova et al., 2007; Barriopedro et al., 2019). Conversely, not finding any benefit in retiring from sport would manifest itself in a less positive emotional reaction upon retirement and more pressing transition demands (Stambulova et al., 2007). By the same token, we assumed voluntariness as well as timeliness of career termination to be beneficial with respect to both emotional reactions and extent of adjustment after ending one’s career (Cecić Erpič et al., 2004; Stambulova et al., 2007).

Empirical studies have used many ways of operationalizing successful transitions out of elite sport, which differ mainly with respect to temporal scale and thematic specificity (Barth et al., 2021). These outcomes include duration of the adaptation to athletic retirement (Grove et al., 1997), perceived success in coping with retirement (Stambulova et al., 2007), symptoms of anxiety or depression (van Ramele et al., 2017), life satisfaction (Shander and Petrie, 2021), subjective well-being (e.g., Stephan et al., 2003b), and self-esteem (e.g., Kleiber and Brock, 1992). Most often, the extant literature does not justify why a particular outcome has been chosen, indicating that these constructs are perceived to be essentially interchangeable or mere facets of general well-being. In the present study, we focus on self-esteem, as it “is a causal force that influences people’s lives in the domains of relationships, work, and health” (Orth and Robins, 2019, p. 338).

Returning to Figure 2, we hypothesized that the two short-term consequences of career termination, emotional reactions upon retirement and the extent of adjustment, predict both the duration and the quality of the adjustment, which in turn affect self-esteem. Specifically, we expected that the greater the extent of adjustment, the longer the duration and the lesser the quality of adjustment, wherefore more positive emotional reactions would predict a shorter duration and better quality of adjustment.

Although emotional reactions and the extent of adjustment are results of distinct psychological processes, emotional and cognitive, the two constructs were conceptualized to be correlated. In like manner, quality and duration of adjustment were hypothesized to be intertwined, because, for example, the evaluation of the quality of the adjustment may be contingent on the time it took to complete it. However, as no logical ordering of the two constructs can be established, another covariance was incorporated in the model.

Figure 2 contains self-esteem at the first measurement (*t*_1_), for two reasons: first, self-esteem (*t*_1_) represents a resource that acts as a buffer for the self, providing protection from harmful experiences (Cast and Burke, 2002). Thus, self-esteem was assumed to reduce the perceived extent of adjustment and have a positive indirect effect on the medium- and long-term influence of athletic retirement. Second, based on the relative stability of self-esteem in adulthood (Wagner et al., 2016), it was expected that self-esteem (*t*_1_) directly influences self-esteem at the second measurement (*t*_2_).

To reduce clutter in Figure 2, associations between the identified preconditions and transition characteristics have been omitted. However, following common practice in SEM, they are estimated. Also excluded from Figure 2 is the time between athletic retirement and the second survey. Several studies reported positive associations between the time since retirement and the quality of the adjustment process (e.g., Stephan et al., 2003b). Nevertheless, when analyzing the final model, we take the time since athletic retirement into consideration because in this regard great inter-individual differences must be anticipated in the sample.

## 2. Materials and methods

### 2.1. Participants and procedure

The study is based on a whole-population survey among the 903 German-speaking (junior) elite athletes who were carded at *t*_1_ by the Swiss Olympic Association. Names and addresses, to which a paper-and-pencil survey was sent, were obtained from the Swiss Olympic Association. At *t*_2_, 12  years later, we were able to retrieve the addresses of 825 of these (now mostly former) athletes (91%). For this purpose, individual federations were contacted, and a particular address-finding service of the Swiss Post was used. Overall, the search lasted several months (for a detailed description of the recruitment process, see Engel, 2014). At their discretion, the questionnaire was administered using either a paper-and-pencil or an online format. Prior to both surveys, a letter of motivation to participate was sent to the athletes by the Swiss Olympic Association. However, participation in the study was voluntary. At the time of data collection, ethical review and approval was not required for the study on human participants according to local legislation and institutional requirements. The study was conducted in accordance with the Declaration of Helsinki (World Medical Association, 2013).

At *t*_1_, 611 athletes (68% of the population) and at *t*_2_, 417 (former) athletes (46%) returned the questionnaire (with up to three personal reminders). We could include 290 individuals (32% of the population) in the present study who had participated on both occasions and had retired from elite sports before *t*_2_ (see Table 1). The sample comprised of 95 women and 195 men from 64 different sports. The sample started performance-oriented training at an average age of 13.1 years (*SD* = 5.4) and ended their athletic career at a mean age of 29.4 years (*SD* = 5.9). The average age of the sample was 25.0 years at *t*_1_, and 36.8 years (*SD* = 6.0) at *t*_2_. Upon completion of the second survey, the participants had been retired from elite sport for 7.3  years (*SD* = 3.3) on average.

**Table 1:** Descriptive statistics of respondents (sample) and nonrespondents.

Variable	Respondents (*N* = 290)		Nonrespondents (*N* = 613)		Effect size^a^	*p*
Gender, *n*, %					0.05	0.137^b^
Female	95	32.8	170	27.7		
Male	195	67.2	443	72.3		
Performance level, *n*, %					0.03	0.679^c^
Elite athletes	31	10.7	60	9.8		
Pre-elite athletes	184	63.4	407	66.4		
Junior elite athletes	75	25.9	146	23.8		
Sport category, *n*, %					0.02	0.558^b^
Individual sports	184	63.4	376	61.3		
Team sports	106	36.6	237	38.7		
Age, *M*, *SD*	24.99	6.01	25.56	7.28	0.08	0.215^d^

a Values represent Cramér’s *V* in the case of categorical variables and Cohen’s *d* for continuous variables. The guidelines proposed by Cohen (1988) for Cramér’s *V* are: 0.10 = small, 0.30 = moderate, 0.50 = large effect. Regarding *d* the criteria are 0.20, 0.50, and 0.80.

b Values of *p* were determined by Fisher’s exact test of independence (two-sided).

c The values of *p* was determined by the Fisher–Freeman–Halton exact test of independence (two-sided).

d The value of *p* was determined by the *t* test (two-sided).

To identify potential (self-)selection effects, the completed sample of 290 participants was compared regarding demographic and sport-related characteristics with the remaining 613 (former) athletes of the population, who chose not to respond to one or both surveys or were unable to be contacted. Particularly, a direct binary logistic regression on the probability of participating in the study was conducted to determine the relative importance of gender, age, type of sport (individual vs. team), and performance level (elite, pre-elite, and junior elite athletes). The model containing five independent variables was not significant, *χ*^2^ = 4.178, *N* = 903, d*f* = 5, *p* = 0.524; *R*^2^ (Nagelkerke) = 0.006, indicating that at least with respect to the available information the sample is a good representation of the population.

### 2.2. Measures

In accordance with the constructs delineated in Figure 1, participants were asked to provide data on three topics: preconditions of the transition out of elite sport (*t*_1_); transition characteristics (*t*_2_); and adjustment to, and short-, medium-, and long-term consequences of, career termination (*t*_2_). The measures used in this study and the relevant information regarding variable coding and time of measurement (while the athletes were still active in elite sport vs. after the athletes have terminated their athletic career) are summarized in Table 2. Regarding the preconditions of the transition out of elite sport, validated instruments were available to measure sporting career satisfaction, athletic identity, and self-esteem. Concerning the transition characteristics and the adjustment to, and long-term consequences of, career termination, we relied on items that have previously been used in empirical studies, notably in the European perspectives on athletic retirement project (EPAR; Alfermann et al., 2004; Alfermann and Stambulova, 2007). Besides, Table 2 contains information with respect to the measurement of sporting career success.

**Table 2:** Overview of study variables, instruments, and point in time of assessment.

Construct	Measure	Item count	Time of assessment
Preconditions of Career Termination (CT)	
Sporting career success	The measurement of objective sporting career success was formed on research by De Bosscher et al. (2009) on international sporting success. Specifically, subjects listed all major international competitions in which they took part and the results they achieved therein (for many sports, Olympic Games, World and European Championships). These data were linked with publicly available information on the number of major competitions subjects could have taken part while active in elite sport. This approach translated into a variable reflecting a subject’s aggregated market share which considers the importance of a competition in a particular sport, its frequency, and the associated chances of taking part therein. High values (5) indicated a successful career, low values (1) a less successful one.	–	*t*_2_
Sporting career satisfaction	As a subjective measure of athletic success during a sports career, the *Retrospective Life Satisfaction* scale from the *Life Satisfaction Scale* (LSS; Ferring et al., 1996) was used. Reference in the selected items to “life” in general was changed to “life in sport”. 7-point rating scale (1 = *disagree* to 7 = *agree*). McDonald’s (1999) *ω* = 0.66.	3	*t*_1_
Athletic identity	German version of the *Athletic Identity Measurement Scale* (Brewer et al., 1993; Schmid and Seiler, 2003). 7-point rating scale (1 = *disagree* to 7 = *agree*). McDonald’s (1999) *ω* = 0.82.	7	*t*_1_
Self-esteem	*Bern Questionnaire on Subjective Well-being* (Grob, 1995). 6-point rating scale (1 = *disagree* to 6 = *agree*)	3	*t*_1_, *t*_2_
Transition characteristics (Alfermann et al., 2004; 6-point rating scales)	
Voluntariness of CT	Subjects’ rating of CT as “voluntary” vs. “involuntary”	1	*t*_2_
Timeliness of CT	Subjects’ rating of CT as “too early” vs. “too late”	1	*t*_2_
Retirement planning	Subjects having “sound plans for the time after CT” vs. not having such plans	1	*t*_2_
Gain–loss perception	Subjects’ rating of CT as a loss” vs. “a gain”	1	*t*_2_
Short- and medium-term adjustment to, and consequences of, career termination	
Emotional reactions	Emotions are considered as an immediate reaction to a subject’s assessment of the transition demands (Asendorpf, 2007). Positive emotions were focused upon, because positive rather than negative ones promote adaptation and well-being (Helgeson et al., 2006). Subjects reported retrospectively their reactions to CT in terms of happiness, joy, freedom/independence, and relief. 5-point rating scale (1 = *no*, 2 = *rather no, …*, 5 = *yes*; Alfermann et al., 2004).	4	*t*_2_
Extent of adjustment	Subjects’ self-reported extent to which CT introduced a discontinuity in their life and necessitated adjustment in professional, financial, social, and bodily terms (1 = *no adjustment* to 5 = *major adjustment*; Wylleman et al., 2011; Gilmore, 2008)	4	*t*_2_
Duration of adjustment	Self-reported time it had taken participants to adjust to CT (Alfermann et al., 2004; variable coded in years)	1	*t*_2_
Quality of adjustment	Self-reported degree to which subjects succeeded to adjust to CT (1 = *very poorly* to 7 = *very well*; adapted from Grove et al., 1997)	1	*t*_2_

CT, career termination. In the case of all constructs, a high score indicated a high degree of the quality that is suggested by the construct name. For example, a high score in the items or scales capturing quality of adjustment or self-esteem reflected a high quality of adjustment and a high level of self-esteem, respectively. Similarly, in all characteristics of CT, high scores stood for an event-related property that is usually perceived to be a transition resource, while low scores stood for transition barriers.

### 2.3. Statistical analyses and data analyses strategy

SEM was the main statistical procedure applied to test the hypothesized model (see, e.g., Kline, 2016). These analyses were conducted using the R package lavaan (version 0.6–8, Rosseel, 2012), while IBM SPSS Statistics (Version 28) was used for all other statistical analyses.

With respect to the 33 variables of interest in the dataset, there were complete data from 208 participants, while the remaining 82 cases accounted for the 1.7% of the missing data. Incomplete reporting was mainly related to the duration (13.8%) and the quality of adjustment to career termination (9.7%), and the emotional reactions to it (freedom: 4.8%; happiness: 4.1%). Missing values were singly imputed using the expectation–maximization algorithm through SPSS MVA.

Screening the data for univariate outliers resulted in five cases with an unusually long time of adjustment to life after sport or an impossibly long time since career termination. These cases remained in the sample. However, they were assigned a raw score on the offending variables that was one unit larger than the next most extreme, but plausible case in the sample (Tabachnick and Fidell, 2013). With the use of a *p* < 0.001 criterion for Mahalanobis distance based on all variables in the initial SEM model, two multivariate outliers with low values on the self-esteem variables (*t*_2_) were identified. In view of the small number, the two cases were kept in the analyses, too.

Analyses of the assumptions underlying SEM indicated that a normal multivariate distribution of the model variables could not be assumed. Therefore, robust maximum-likelihood estimation was used. It provides a scaled test statistic that is (asymptotically) equal to the Yuan–Bentler test statistic and robust (Huber–White) standard errors. Unit loading identification was used to set the metric of the latent variables.

Based on Kline (2016), the following fit statistics were used to assess the quality of SEM models: the Comparative Fit Index (CFI), the Root Mean Square Error of Approximation (RMSEA), and the Standardized Root Mean Square Residual (SRMR). We considered values of CFI ≥ 0.95, RMSEA ≤ 0.06, and SRMR ≤ 0.08 to indicate excellent fit, and values of CFI ≥ 0.90, RMSEA ≤ 0.08, and SRMR ≤ 0.10 to reflect adequate fit. The Satorra–Bentler-Scaled-*χ*^2^-difference-test was used to compare the fit of multiple models. However, we focused on the CFI and RMSEA difference between models (Chen, 2007). If it exceeded 0.010 or 0.015, respectively, the null hypothesis of equal fit was rejected. Indirect effects were estimated by the product of the coefficient of each of the paths in the mediational chain and tested using the percentile bootstrap method (MacKinnon, 2008).

SEM analyses followed a two-step approach (e.g., Kline, 2016): first, using confirmatory factor analysis (CFA), a model with all latent variables was fitted to the data to ensure that the measurement models hold. Second, we investigated the proposed model (Figure 2). Given the preliminary nature of this model, we expected some degree of model misspecification and a need to probe into the model by adding or reducing complexity as might be the case. In this follow-up, SEM was applied in a more exploratory way. Expected parameter changes and modification indices were assessed using the methods proposed by Saris et al. (2009) and implemented in the R package semTools (Jorgensen et al., 2021). Unfortunately, the order in which parameters are freed or restricted can affect the significance of the remaining parameters. Following Tabachnick and Fidell (2013) recommendation, all necessary parameters were added before unnecessary ones were removed. Since new data were not available for cross-validation, we computed the correlation between the estimated parameters from the hypothesized and the final model using parameters common to both models. If this correlation was close to 1, we concluded that relationships within the model have been retained notwithstanding the *post hoc* modifications. Results of an *a posteriori* power analysis are provided in the results section.

### 2.4. Transparency and openness

We have cited all data, program codes, and other methods developed by others appropriately in the text and listed them in the reference section. Furthermore, criteria for participants to be included or excluded in the corpus of data are detailed as well as all measures used in the study. The data are not available publicly because sports results and other variables (e.g., year of career end) are freely accessible and give clear indication of the identity of the study participants. Data and questionnaires may be made available by contacting the corresponding author. The design of the study and its analysis were not pre-registered.

## 3. Results

### 3.1. Descriptive statistics, correlations, and fit statistics

The means, standard deviations and correlations between all variables used for SEM are reported in Table 3; values of selected fit statistics for all estimated models are presented in Table 4. On a descriptive level, it is worth mentioning that the athletes indicated a relatively high need for occupational and bodily adjustment after retiring from elite sport. They also reported that, on average, it took them 11.99 months (*SD* = 10.62) to complete the process of adjustment. From a subjective point of view, 2.8% of the athletes described the quality of their adjustment as very poor to rather poor, 4.8% as middling, and 92.5% as rather good to very good.

**Table 3:** Zero-order correlations, means, standard deviations, and percentage of missing data for variables in the model.

	Variable	1	2	3	4	5	6	7	8	9	10	11	12	13	14	15	16	17	18	19	20	21	22	23	24
	Preconditions of career termination																								
	Self-esteem (*t*_1_)																								
1	se1	–																							
2	se3	0.60	–																						
3	se3	0.42	0.59	–																					
4	success	0.05	0.09	0.03	–																				
5	satis	0.14	0.17	0.18	0.14	–																			
6	ai	0.04	−0.05	−0.09	0.02	0.10	–																		
	Characteristics of career termination																								
7	vlnt	−0.08	−0.02	0.00	0.10	0.06	−0.05	–																	
8	tmln	−0.13	−0.11	0.00	0.14	−0.01	−0.08	0.34	–																
9	gnlss	−0.06	0.05	0.13	0.02	−0.02	−0.11	0.33	0.37	–															
10	plnn	0.04	0.01	0.07	−0.10	0.04	−0.03	0.20	0.20	0.26	–														
	Short-, medium- and long-term adjustment and consequences																								
	Extent of adjustment																								
11	eoa1	0.09	0.07	0.06	0.04	0.01	0.21	0.06	−0.08	−0.01	−0.20	–													
12	eoa2	0.01	0.01	0.04	0.00	−0.02	0.22	0.02	0.00	−0.04	−0.14	0.53	–												
13	eoa3	−0.04	−0.06	−0.12	0.09	0.03	0.13	−0.05	−0.14	−0.14	−0.17	0.28	0.18	–											
14	eoa4	0.02	0.08	0.01	0.01	0.02	0.14	−0.20	−0.17	−0.25	−0.24	0.09	0.07	0.30	–										
	Emotional reactions																								
15	er1	−0.06	0.00	−0.01	0.04	−0.06	−0.03	0.39	0.32	0.43	0.06	0.03	−0.02	−0.03	−0.12	–									
16	er2	−0.01	0.03	0.14	0.09	0.01	0.00	0.40	0.35	0.47	0.18	0.01	−0.01	−0.11	0.49	0.49	–								
17	er3	−0.05	0.02	0.11	0.11	0.00	−0.07	0.48	0.39	0.48	0.19	−0.06	−0.06	−0.09	0.57	0.57	0.73	–							
18	er4	−0.06	0.04	0.03	0.03	−0.03	0.05	0.25	0.27	0.34	0.12	−0.12	−0.10	−0.02	0.58	0.58	0.41	0.46	–						
19	adjqual	0.03	0.11	0.27	−0.08	0.11	−0.05	0.15	0.15	0.22	0.19	−0.07	0.05	−0.14	−0.01	−0.01	0.18	0.20	0.10	–					
20	adjdur	0.06	−0.03	−0.08	−0.03	0.01	0.13	−0.16	−0.24	−0.34	−0.14	0.23	0.12	0.32	−0.21	−0.21	−0.25	−0.35	−0.24	−0.35	–				
	Self-esteem (*t*_2_)																								
21	se1	0.26	0.20	0.07	−0.08	−0.05	0.01	−0.08	−0.08	0.04	−0.01	0.14	0.06	0.06	−0.02	−0.02	−0.06	−0.05	−0.02	0.18	0.03	–			
22	se2	0.23	0.30	0.18	−0.03	0.04	0.01	−0.11	−0.02	−0.03	0.01	0.03	−0.07	0.03	0.02	0.02	0.04	0.00	0.02	0.17	0.00	0.52	–		
23	se3	0.17	0.25	0.29	−0.05	−0.01	−0.07	−0.07	0.00	0.09	0.05	−0.03	−0.01	−0.09	−0.03	−0.03	0.12	0.06	0.02	0.20	−0.06	0.38	0.70	–	
	Control variable																								
24	time	0.01	−0.04	−0.07	0.01	−0.02	−0.19	−0.05	−0.10	−0.05	−0.10	−0.01	0.05	0.05	−0.08	−0.08	0.03	0.01	−0.06	0.06	0.07	−0.04	0.00	−0.03	–
	*M*	5.17	5.38	5.23	2.67	3.26	5.36	4.98	1.52	3.87	4.31	2.99	2.08	2.47	2.67	2.99	2.69	2.83	3.57	6.20	1.00	5.18	5.26	5.17	7.16
	*SD*	0.73	0.74	0.83	1.50	0.49	0.95	1.60	0.74	1.24	1.67	1.57	1.26	1.23	1.23	1.28	1.16	1.19	1.34	1.06	0.89	0.71	0.70	0.77	3.32
	Missing (%)	0.0	0.3	0.0	0.0	1.0	1.4	1.4	2.1	1.4	1.0	1.4	2.1	2.1	2.1	1.4	4.1	2.1	4.8	9.7	13.8	0.7	0.3	0.3	2.8

*N* = 290. Correlations ≥ |0.15| are significant at the 5% level; correlations ≥ |0.14| are significant at the 1% level (two-sided test). *t*_1_/*t*_2_, first/s point of measurement; success, sport career success; satis, sport career satisfaction; ai, athletic identity; vlnt, voluntariness; tmln, timeliness; gnlss, gain–loss; plnn, retirement planning; eoa, extent of adjustment (eoa1, vocational; eoa2, financial; eoa3, social; eoa4, bodily); er, emotional reactions (er1, relief; er2, happiness; er3, joy; er4, independence/freedom); adjqual, quality of adjustment; adjdur, duration of adjustment (years); time, elapsed time since career termination.

**Table 4:** Fit statistics for different structural equation models.

	Model description	d*f*	MLR *χ*^2^	*p*	CFI	RMSEA	95% CI	SRMR	AIC	BIC
	Measurement (CFA) models									
A1	Base CFA model	158	313.143	<0.001	0.908	0.059	[0.049, 0.068]	0.055	17893.633	18411.086
A2	A1 with correlated errors for a pair of indicators of Emotional Reactions (relief ~ ~ freedom)	157	275.089	<0.001	0.930	0.052	[0.041, 0.062]	0.054	17856.875	18377.998
A3	A2 with correlated errors for a pair of indicators of Extent of Adjustment (financial ~ ~ vocational)	156	241.511	<0.001	0.949	0.044	[0.033, 0.054]	0.045	17822.567	18347.360
	Structural regression models									
B1	Theoretical model (as diagrammed in Figure 1)	181	274.778	<0.001	0.944	0.043	[0.032, 0.053]	0.051	17810.071	18243.117
B2	B1 with added regression path (Quality of Adjustment ~ Sport Career Satisfaction)	180	270.463	<0.001	0.946	0.042	[0.030, 0.051]	0.050	17807.285	18244.000
B3	B1 with added regression path (Quality of Adjustment ~ Self-Esteem at *t*_1_)	180	269.636	<0.001	0.947	0.042	[0.031, 0.052]	0.049	17805.936	18242.652
B4	B1 without 12 non-significant regression paths	193	284.667	<0.001	0.945	0.041	[0.031, 0.051]	0.053	17796.750	18185.757
B5	B4, MIMIC-model to control for the association between the exogenous manifest variable time since career termination and all exogenous and endogenous latent variables (5 regression paths and 8 covariances added)	203	306.866	<0.001	0.939	0.043	[0.033, 0.052]	0.052	19322.536	19766.592

*N* = 290. MLR = robust maximum-likelihood estimation with a scaled test statistic that is (asymptotically) equal to the Yuan–Bentler test statistic; CFI, (robust) comparative fit index; RMSEA, (robust) root mean square error of approximation; 95% CI, 95% confidence limits for RMSEA. SRMR, standardized root mean square residual. Comparison of models: A3 vs. A2: Δ*χ*^2^(1) = 44.877, *p* < 0.001; A4 vs. A3: Δ*χ*^2^(1) = 16.475, *p* < 0.001; B2 vs. B1: Δ*χ*^2^(1) = 3.820, *p* = 0.051; B3 vs. B1: Δ*χ*^2^(1) = 3.869, *p* = 0.049. B4 vs. B1: Δ*χ*^2^(12) = 10.084, *p* = 0.609. Comparison of model B4 and B1 with respect to all 41 common major structural parameter estimates (path coefficients and latent covariances): *r* > 0.99, d*f* = 39; maximum (absolute) difference of parameters = 0.06. Comparison of model B5 and B4 with respect to all 41 common major structural parameter estimates (path coefficients plus latent covariances): *r* > 0.99, d*f* = 40; maximum (absolute) difference of parameters = 0.02.

### 3.2. Measurement models

CFA were performed with 13 latent constructs (and 23 indicators), which could covary freely. Self-esteem was indexed by three items. Emotional reactions was covered by four indicators, namely, “relief,” “happiness,” “joy,” and “independence/freedom,” as was Extent of Adjustment (“vocational,” “financial,” “social,” and “bodily”). Given our medium sample size and in order not to add more complexity to the model, we chose to model both sporting career satisfaction and athletic identity as a single-indicator factor (Savalei, 2019) by averaging the three and seven item scores belonging to each factor. For each factor the directional path was set to one, and its error variance was fixed to $\left(1-r_{\mathrm{XX}\prime }\right)\;\;.\;\;\sigma_{X}^{2}$, where $r_{\mathrm{XX}\prime }$ and $\sigma_{X}^{2}$ are the variable’s reliability (McDonald’s, 1999, coefficient *ω*, see Table 2) and variance, respectively. The remaining latent factors—Sporting Career Success, the four transition characteristics, as well as Duration of Adjustment and its Quality—were also represented by a single-indicator factor with its reliability arbitrarily fixed to 0.90.

The fit of the basic CFA model A1 with 13 latent variables was not satisfactory, *χ*^2^(158) = 313.14, *p* < 0.001; CFI = 908; SRMR = 0.055; RMSEA = 0.059. Inspection of the residuals and modification indices suggested to allow an error covariance between two indicators of the factor Emotional Reactions and between two indicators of extent of adjustment. As the emotional reactions of relief and freedom/independence share the idea that the career termination resembles a lift-off of a burden, which the other indicators (joy, happiness) do not cover, there seemed justifiable grounds for specifying correlated errors (model A2). Additionally, it seemed reasonable that the indicators of occupational and financial adjustment both touch upon monetary aspects and thus have closer theoretical ties than the other two items measuring social and bodily adjustment. Hence, model A3 allowed the error variance between the two indicators to be freely estimated. The fit of both models improved significantly, A2 vs. A1: Δ*χ*^2^(1) = 44.877, *p* < 0.001; A3 vs. A2: Δ*χ*^2^(1) = 16.475, *p* < 0.001. As the fit statistics were favorable for model A3 (CFI = 0.949; SRMR = 0.045; RMSEA = 0.044) and local fit testing did not suggest otherwise, model A3 was retained for further analysis.

The standardized loadings on the factors Self-Esteem (at *t*_1_ and *t*_2_) and Emotional Reactions ranged from 0.52 to 0.95. The loadings on Extent of Adjustment, however, were lower (0.27 < *λ* < 0.55). This may be attributed to the ad-hoc nature of the factor, but it can also be seen as an indication of a relatively broad construct.

### 3.3. Structural models

In the second step of analysis the postulated structural regression model B1 (Figure 2) was estimated and found to produce an acceptable fit, *χ*^2^(181) = 274.778, *p* < 0.001; CFI = 0.944, SRMR = 0.051, RMSEA = 0.043. The next step was to improve, if possible, the hypothesized model B1: to this aim, we conducted specification searches based on expected parameter changes and modification indices. Guided by the criteria of Saris et al. (2009) and theoretical considerations, two additional paths were sequentially added to model B1 and tested: a direct effect of both Sporting Career Satisfaction as well as Self-Esteem (*t*_1_) on Quality of Adjustment (model B2 and B3). Based on our evaluation criteria, namely, model fit, chi-square difference test, magnitude, and significance of regression parameter, neither model B2 nor B3 was invariably better than model B1 and were thus rejected. In view of the problems that occur when models are respecified on empirical, albeit not entirely atheoretical grounds approach (Kline, 2016), no new parameters were added the model.

As model B2 is rather complex, we examined it with an eye to the presence of empirically unnecessary directional paths. Inspection of the univariate Wald tests and the absolute magnitude of the expected parameter changes revealed that 12 directional paths among the latent variables may not be necessary. The fit statistics revealed that this restricted model B4 fit as well as model B2 to the data, *χ*^2^(193) = 284.667, *p* < 0.001; CFI = 0.945, SRMR = 0.053, RMSEA = 0.040; B4 vs. B1 Δ*χ*^2^(12) = 10.084, *p* < 0.609. Favoring parsimonious models, we chose model B4 to be the final one.

Because model B4 had been modified *post hoc*, evidence was sought that the hypothesized model has not changed substantially. As no data of an independent sample was available for cross-validation, we carried out a sensitivity analysis (Byrne et al., 1989): the correlation between the major parameters (i.e., regression weights and latent covariances) of the structural regression in model B4 with those in model B1 was estimated at >0.99 (d*f* = 39), suggesting that the relative magnitude of the estimated parameters was not affected by the modification of the model. The final model B4 with significant standardized and unstandardized coefficients is diagrammed in Figure 2.

In terms of the direct effects, Figure 2 shows that Self-Esteem after career termination was predicted by the first measurement of Self-Esteem (stand. Est. *γ* = 0.34, *p* < 0.001). More importantly, Self-Esteem after the end of the career was also predicted by the Quality of the Adjustment to career termination (stand. Est. *β* = 0.16, *p* = 0.016), but not by the time it took to adjust to it. Duration and Quality of Adjustment were significantly associated (*r* = −0.23, *p* = 0.007), and both medium-term consequences of career termination were predicted by Extent of Adjustment: the more areas were in need of adjustment or the greater the necessary adaptation was, the longer it took to adjust (*β* = 0.47, *p* < 0.001); and vice versa, the smaller the Extent of Adjustment was, the better the Quality of the Adjustment was perceived (*β* = −0.37, *p* < 0.001). Emotional reactions to career termination did not predict Quality of Adjustment, but Duration of Adjustment, with more positive Emotional Reactions predicting a shorter Duration of Adjustment (*β* = −0.24, *p* < 0.001).

Emotional Reactions were not linked with Retirement Planning, but with the other transition characteristics: Voluntariness and Timeliness of the career end were associated with more positive emotions (*β* = 0.35, *p* < 0.001, and *β* = 0.17, *p* = 0.009, respectively), as was the case with perceiving the ending of one’s career to be more a gain than a loss (*β* = 0.40, *p* < 0.001). Also, Extent of Adjustment decreased with the degree of Retirement Planning (*β* = −0.33, *p* < 0.001), the extent to which career termination was perceived as a gain (*β* = −0.25, *p* = 0.011), and the narrowing of one’s identity to the athletic role (*β* = 0.28, *p* = 0.001). However, Sporting Career Success and Sporting Career Satisfaction, which are two components of the preconditions in the model, had no direct effect on the adjustment to, and the consequences of, career termination, whether short-, medium- or long-term.

The postulated model also posits indirect effects of preconditions of career termination and transition characteristics, although they have not often been of explicit interest to the empirical research in the field. As can be seen in Figure 2, three different three-path mediations were analyzed, namely potential effects from a thletic Identity, Gain–Loss, and Retirement Planning through Extent of Adjustment (first mediator) and Quality of Adjustment (second mediator) on Self-Esteem at the second measurement. Unfortunately, interpreting the inferential statistical results of mediation analyses (Table 5) is not straightforward, because results may not coincide depending on whether unstandardized or standardized estimates are used. Based on unstandardized estimates (as favored by MacKinnon, 2008, and Muthén, 2012), all three-path mediations were significant, as the 95% bootstrap confidence intervals of the indirect effects did not include zero. (However, no significance was found when standardized estimates were used.) Thus, former athletes with a pronounced athletic identity reported a greater Extent of Adjustment, which in turn was associated with lower Quality of Adjustment and reduced Self-Esteem, unstand. Est. = −0.013, 95% CI [−0.026, −0.002]. This applies *mutatis mutandis* to former athletes who had fewer plans for their career end, unstand. Est. = −0.008, 95% CI [−0.000, −0.022], as well as former athletes who did not see their career ending as gain, but rather a loss, unstand. Est. = −0.008, 95% CI [−0.001, −0.018].

**Table 5:** Unstandardized and standardized estimates, standard errors, and confidence intervals of indirect effects (model B4).

Indirect effects	Unstandardized			Standardized		
	Estimate	SE	95% CI	Estimate	SE	95% CI
Three-path indirect effects						
Athletic Identity → Extent of Adjustment → Quality of Adjustment → Self-Esteem (*t*_2_)	−0.013	0.006	[−0.026, −0.002]	−0.016	0.015	[−0.045, 0.012]
Gain–Loss → Extent of Adjustment → Quality of Adjustment → Self-Esteem (*t*_2_)	0.008	0.006	[0.000, 0.022]	0.014	0.015	[−0.014, 0.043]
Retirement Planning → Extent of Adjustment → Quality of Adjustment → Self-Esteem (*t*_2_)	0.008	0.004	[0.001, 0.018]	0.019	0.017	[−0.015, 0.053]
Two-path indirect effects						
Voluntariness → Emotional Reactions → Duration of Adjustment	−0.047	0.014	[−0.077, −0.022]	−0.085	0.027	[−0.137, −0.032]
Timeliness → Emotional Reactions → Duration of Adjustment	−0.049	0.024	[−0.099, −0.008]	−0.041	0.020	[−0.080, −0.002]
Gain–Loss → Emotional Reactions → Duration of Adjustment	−0.069	0.024	[−0.124, −0.029]	−0.097	0.035	[−0.167, −0.280]

The standardized solution is completely standardized. CI, confidence interval. The percentile method was used for estimating bootstrapped confidence intervals for the indirect effects (5,000 bootstrap samples).

Moreover, three of the four transition characteristics were found to affect the Quality of Adjustment through positive Emotional Reactions (Table 5). More precisely, former athletes who described their career ending as voluntary, as timely or as involving a role gain rather than role loss experienced more positive emotions as an immediate response to the end of their athletic career, which in turn was associated with a shorter duration of adjustment. Again, the confidence interval of all three indirect effects did not include zero, Voluntariness: unstand. Est. = −0.05, 95% CI [−0.08, −0.02]; Timeliness: −0.05 [−0.10, −0.01]; Gain–Loss: −0.07 [−0.12, −0.03].

To avoid clutter, we have not included covariances between the exogenous latent variables in Figure 2, but they are reported in Table 6. Noteworthy correlations (*r* > |0.30|, *p*s < 0.05) were found among the transition characteristics, for example, between Timeliness and Voluntariness (*r* = 0.38) or Timeliness and Gain–Loss (*r* = 0.41). The factors pertaining to the preconditions of career termination, however, were more loosely related to the other factors. Nevertheless, increases in Sporting Career Satisfaction were linked with higher values on Self-Esteem (*t*_1_, *r* = 0.25), and (objective) Sporting Career Success correlated with (subjective) Sporting Career Satisfaction (*r* = 0.18). Athletic Identity, however, was independent of the other preconditions of career termination in the model.

**Table 6:** Correlations between latent exogenous variables (model B4).

	Latent variable	1	2	3	4	5	6	7	8
Preconditions of career termination									
1	Sport Career Satisfaction	–							
2	Athletic Identity	0.14	–						
3	Sport Career Success	0.18*	0.02	–					
4	Self-Esteem (*t*_1_)	0.25*	−0.05	0.09	–				
Characteristics of career termination									
5	Voluntariness	0.08	−0.05	0.12	−0.04	–			
6	Timeliness	−0.01	−0.10	0.16*	−0.12*	0.38*	–		
7	Gain–Loss	−0.03	−0.13*	0.02	0.05	0.36*	0.41*	–	
8	Retirement Planning	0.05	−0.03	−0.11	0.03	0.22*	0.23*	0.29*	–

*N* = 290. *p < 0.05 (two-sided).

About 14% of the variance in Self-Esteem (*t*_2_) was accounted for by the model. The proportion of explained variance in Quality of Adjustment was 14%, while the values for Duration (*R*^2^ = 0.34), Extent of Adjustment (*R*^2^ = 0.32), and Emotional Reactions (*R*^2^ = 0.51) were markedly higher.

### 3.4. Additional analyses

To control for the potential confounding effect induced by individual differences regarding the time that has elapsed between career termination and the second measurement, time was included as a covariate in what amounts to a multiple indicators multiple independent causes (MIMIC) model. Thus, compared with model B4, in model B5 the five endogenous latent variables Emotional Reactions, Extent, Quality, and Duration of Adjustment, and Self-Esteem (at *t*_2_) as well as the exogenous latent variables (preconditions and transition characteristics) were regressed on the variable time since career termination. The fit statistics for model B5, *χ*^2^(203) = 306.866, *p* < 0.001, CFI = 0.939, SRMR = 0.052, RMSEA = 0.043, revealed a negligible drop in comparison with model B4.

To estimate the *a posteriori* statistical power on the model level, we used a method by MacCallum et al. (1996), which is based on the RMSEA and implemented in semTools. In essence, the analysis revealed that the probability of detecting a model with “good” approximate fit was well above traditional power values of 0.80.

## 4. Discussion

Working on the notion that career termination in elite sport is a complex and dynamic process which is subdivided into a temporal, phase-like structure and is best analyzed from a broad perspective using a holistic lifespan perspective (e.g., Debois et al., 2015; Wylleman, 2019), the present study employed a holistic model to provide a better understanding of how interindividual differences in athletic retirement and self-esteem in life after elite sport are linked. Our goal was to examine the interplay of some of factors which have emerged in the empirical literature and their long-term effect on self-esteem in life after elite sport. Specifically, we analyzed the relations between preconditions of career termination, transition characteristics, adjustment to career termination, and consequences on self-esteem.

SEM analyses revealed that many, but not all hypothesized relations were compatible with our data. Under statistical control of all other variables, Sporting Career Success and Sporting Career Satisfaction did not affect short-, medium-, or long-term adjustment directly. However, Retirement Planning (e.g., for socio-professional reinsertion) and Athletic Identity predicted Extent of Adjustment to career termination, which in turn, via Duration and Quality of Adjustment, ultimately predicted Self-Esteem. Voluntariness, Timeliness, and perceptions of Gain vs. Loss predicted Emotional Reactions to career termination and Duration of Adjustment. Extent of Adjustment and Emotional Reactions mediated between preconditions of career termination and transition characteristics and Self-Esteem. The final model explained 14% of the variation in Self-Esteem after career termination. It was predominantly predicted by Self-Esteem 12 years earlier as an athlete (11%, i.e., stand. Est. *γ* squared), but factors related to career termination nevertheless significantly affected Self-Esteem several years after athletic retirement (3%). In the absence of reasonably comparable studies in the field as well as widely accepted interpretative guidelines, an assessment of such an effect remains arbitrary (Sarstedt et al., 2022). Nevertheless, we judge the effect as small.

Five aspects of these findings warrant discussion, which refer to both the entire model as well as specific elements thereof: first, regarding the stability of the trajectory of individuals’ self-esteem, considerable heterogeneity was found. Specifically, self-esteem was clearly less stable over the 12  year period in our study (estimated as 0.34) than in Kuster and Orth’s study (2013) study, who found that long-term stability of self-esteem approached a value of 0.60 for a time interval of 12  years. Thus, there is clear indication that retirement from elite sport is linked with changes in self-esteem, which are still noticeable several years thereafter.

Second, with regard to the amount of explained variance in Self-Esteem, one might have expected a larger effect. But it needs to be kept in mind that this is the effect after statistical control of Self-Esteem at *t*_1_ and, above all, that there are factors in life domains such as social relationships, work or health that might have a greater effect on self-esteem than a temporally more or less remote career in elite sport (Reitz, 2022). Hence in the long run, athletic retirement does not appear to be the tragic event, as was assumed in the early research in this area (e.g., retirement as social death; Rosenberg, 1982).

Third, looking at the antecedents of Self-Esteem (*t*_2_) in the model, we recognize that the results are in line with much of the empirical literature and thus corroborate many of the (bivariate) associations between preconditions and transition characteristics and consequences of athletic retirement. Although not novel, the results are valuable because they were gained using a data-analysis technique which allows to control not only for measurement error but for confounding variables as well (e.g., Kline, 2016). Control for confounding might well be the reason why several hypothesized associations in the model were not supported by the data (dotted arrows in Figure 2). For example, regarding Voluntariness and Retirement Planning we found that they did not affect Extent of Adjustment and Emotional Reactions, respectively, over and above the effects of the other variables in the model, notably the two transition characteristics of career termination, Timeliness and Gain–Loss. Contrary to earlier research (Cecić Erpič et al., 2004), analyses revealed that Sporting Career Success as well as Sporting Career Satisfaction did not predict the short-term consequences of career termination nor Duration of Adjustment. Besides, Athletic Identity was not found to have a direct effect on Emotional Reactions, Duration or Quality of Adjustment, but rather to have an indirect one, mediated by Extent of Adjustment. Thus, athletes with a strong, exclusive athletic identity are not *per se* at risk, but they are likely to make educational, occupational, financial, or social choices which turn out to be suboptimal for the quality of transition and ultimately self-esteem in life after elite sport. The possibility of testing such mediation effects is related to the use of SEM as an analysis procedure. This technique which is not often used in this field of research, allowed us to draw a more precise and valid picture (Kline, 2016) of the interplay between preconditions of career termination and transition characteristics and its consequences.

Fourth, time since career termination has been included as a covariate in the model. Our goal was to control for the potential confounding effect induced by individual differences regarding the time of retirement. It is interesting to note that there was no significant parameter estimate involving time. Specifically, neither the perceived transition demands nor the emotional reactions upon career termination, nor self-esteem (*t*_2_) was affected by the time since career termination. These results can be interpreted as a lack of evident time-dependent recall bias (although recall bias *per se* might still be present) or that the fading affect bias (Skowronski et al., 2006) is not present when it comes to athletic retirement. This result is supported by other studies in which the time since former elite athletes stopped competing was also not (linearly) related to the occurrence of mental health disorders or subjective well-being (Sanders and Stevinson, 2017; van Ramele et al., 2017). Conversely, qualitative longitudinal studies reported that retired athletes perceived fewer transition difficulties over time (Stephan et al., 2003b; Lally, 2007). However, these studies are not comparable to the present one, because they covered a much shorter time span, up to one year, and therefore address short- or medium-term consequences of career termination.

Fifth, from a conceptual point of view, our model contains a differentiation regarding both time and nature of measurement: in compliance with Stambulova et al. (2021), we attempted to depict the temporal structure underlying the adjustment process and, as a result, differentiated short-, medium- and long-term aspects. We also distinguished between objectively real phenomena and subjectively real ones, for example, duration and perceived quality of adjustment. We believe that this added complexity may also help to better design athlete career support programs. For example, our findings suggest that career assistance programs (for an overview, see Torregrosa et al., 2020), as they are offered by sport federations, should be accessible to former athletes more than one year past career termination. Additionally, career assistance programs should go beyond employment services and include social, emotional, and bodily care programs.

### 4.1. Limitations

The present study has several limitations. First, it was conducted among Swiss (former) athletes, who were, in essence, active in the nineties and noughties. Although our sample might be comparable to samples of other Western European countries, it is likely to differ, for example, from Asian samples (Ling and Hong, 2014). Moreover, the sport system is ever changing and has spurred in recent years professionalization, mediatization, and commercialization of elite sport (Westerbeek and Hahn, 2013). In turn, sport federations have expanded their efforts to support athletes to meet the increased demands (e.g., regarding time commitment), and athletes have started to benefit from the new opportunities in the field of sport (e.g., with respect to work and earnings; Schmid et al., 2022) associated with the recent developments. In view of potential socio-cultural and epochal peculiarities, future studies should aim for better generalizability of the results using samples of retiring athletes from other contexts.

Second, because the project started in the early noughties, measurement instruments were used which were state of-the-art of that time. In the meantime, new instruments have been advanced. Thus, it is recommended to take up these developments and, for example, measure identity multidimensionally in order to better reflect the holistic view on retirement (Yukhymenko-Lescroart, 2014).

Third, although our model is already quite large, additional variables could or should be integrated. These include, amongst others, the reasons for leaving competitive sport (Taylor and Ogilvie, 1994), the retirement decision-making process (Fernandez et al., 2006), strategies for coping with the transition (Stambulova, 2003), and the availability of social support to retired athletes (Stephan et al., 2003b). Without doubt, the model would be more comprehensive if these variables had been considered. Nevertheless, these aspects are not completely neglected in the model because, for example, efficient instrumental coping, a full-time job offer following career termination, or significant social support would reduce the perceived extent of adjustment.

Fourth, cross-validation of the proposed model is needed because exploratory modification has been undertaken during the data analysis process (Chou and Huh, 2012).

Finally, the study results must be considered in light of the fair, but not high response rate (32%). Although respondents did not differ from non-respondents on key demographic and sport-related variables, we do not know if systematic dropout of former athletes occurred and thus if bias has been introduced in the results (e.g., athletes who had difficulties retiring from elite sport).

### 4.2. Implications for future research

The presented study may have methodological implications for future research: so far, quantitative researchers in the field of athletic career termination have largely relied on traditional data-analysis techniques. Now that more complex conceptual models have been developed and that the interplay and temporal dynamic of the multiple causes and multiple outcomes have become of interest to researchers, they might embrace the opportunities which more powerful statistical methods such as SEM offer (e.g., Ntoumanis and Myers, 2016). Advanced data-analysis techniques will gain value if the call for (intensive) prospective longitudinal study designs necessary to examine dynamic processes (Stephan and Demulier, 2008; Park et al., 2013) is to be answered.

Studying a major life event such as career termination and the short-, medium- and long-term effects among elite athletes raises issues which developmental science addresses. In recent years, dynamic-interactionist concepts of development have dominated developmental theorizing (e.g., Lerner, 2006). Moreover, a holistic perspective on development processes has been promoted (Bergman et al., 2003), which has parallels to the literature on career termination (e.g., Wylleman, 2019). A methodological consequence of this point of view is that the traditional variable-oriented approach based on the general linear model has limited applicability and that person-oriented strategies should also be considered (Conzelmann et al., 2023; for an application in sports career research see, e.g., Schmid et al., 2022). Within the person-oriented approach the individual is viewed as a functioning and developing totality that is best described by analyzing patterns of information rather than individual variables (Bergman et al., 2003) allowing peculiarities of each athlete to be taken into account. The foundation for applying the person-oriented approach is laid by variable-oriented studies, which allow the identification of basic influencing factors.

## 5. Conclusion

In the present study, a holistic model was used to better understand the link between athletic retirement, a life-event which is often described as difficult, and self-esteem in life after elite sport. For this purpose, a relatively large representative sample of Swiss elite athletes was studied twice over a period of 12 years. The results obtained by means of SEM indicated that emotional reactions and the extent of adjustment mediated between individual antecedents and characteristics of career termination and self-esteem. While self-esteem after retirement from sport was predicted primarily by self-esteem 12  years earlier, perceived quality of adjustment to career termination was not without influence. Compared to more traditional analysis techniques in previous research (e.g., ANOVA), the use of SEM provides a more valid picture of the interplay between individual antecedents of career termination, transition characteristics and their consequences. This is especially true with regard to the role of athletic identity, retirement planning, and the extent of adjustment. Specifically, it appears that athletic identity does not directly influence adjustment quality, but rather indirectly via retirement planning and the extent of necessary adjustment to changed life situations.

From a practitioner’s perspective, the importance of retirement planning has once again become obvious (see also Park et al., 2013). With good retirement planning (including clear and actionable plans for the post-athletic life), it is possible to keep the extent of adjustment low. This can be achieved, for example, by pursuing a dual career, by developing a perspective for one’s professional future, by engaging in social relationships outside of sports as well, by recovering from possible (career ending) injuries, and by preparing for a physically less active lifestyle changes with its associated bodily changes.

## Data availability statement

The raw data supporting the conclusions of this article will be made available by the authors, without undue reservation.

## Ethics statement

Ethical review and approval was not required for the study on human participants in accordance with the local legislation and institutional requirements. Written informed consent for participation was not required for this study in accordance with the national legislation and the institutional requirements.

## Author contributions

JS: funding acquisition, methodology, and formal analysis. JS, AC, and MS: conceptualization. JS and RE: investigation and data curation. JS and MS: writing—original draft and supervision. JS, AC, RE, AK, and MS: writing–review and editing. All authors contributed to the article and approved the submitted version.

## Funding

This research was supported by the Federal Commission of Sport (ESK) [grant number 10-12] and the Swiss Olympic Association. The funders had no role in study design, data collection and analysis, or publication process. Open access funding by University of Bern.

## Conflict of interest

The authors declare that the research was conducted in the absence of any commercial or financial relationships that could be construed as a potential conflict of interest.

## Publisher’s note

All claims expressed in this article are solely those of the authors and do not necessarily represent those of their affiliated organizations, or those of the publisher, the editors and the reviewers. Any product that may be evaluated in this article, or claim that may be made by its manufacturer, is not guaranteed or endorsed by the publisher.
